# Microbial Cell Factories for Green Production of Vitamins

**DOI:** 10.3389/fbioe.2021.661562

**Published:** 2021-06-17

**Authors:** Yanyan Wang, Linxia Liu, Zhaoxia Jin, Dawei Zhang

**Affiliations:** ^1^School of Biological Engineering, Dalian Polytechnic University, Dalian, China; ^2^Key Laboratory of Systems Microbial Biotechnology, Chinese Academy of Sciences, Tianjin, China; ^3^Tianjin Institute of Industrial Biotechnology, Chinese Academy of Sciences, Tianjin, China; ^4^National Technology Innovation Center of Synthetic Biology, Tianjin, China; ^5^University of Chinese Academy of Sciences, Beijing, China

**Keywords:** vitamins, metabolic engineering, microbial cell factory, chemical synthesis, biosynthesis

## Abstract

Vitamins are a group of essential nutrients that are necessary to maintain normal metabolic activities and optimal health. There are wide applications of different vitamins in food, cosmetics, feed, medicine, and other areas. The increase in the global demand for vitamins has inspired great interest in novel production strategies. Chemical synthesis methods often require high temperatures or pressurized reactors and use non-renewable chemicals or toxic solvents that cause product safety concerns, pollution, and hazardous waste. Microbial cell factories for the production of vitamins are green and sustainable from both environmental and economic standpoints. In this review, we summarized the vitamins which can potentially be produced using microbial cell factories or are already being produced in commercial fermentation processes. They include water-soluble vitamins (vitamin B complex and vitamin C) as well as fat-soluble vitamins (vitamin A/D/E and vitamin K). Furthermore, metabolic engineering is discussed to provide a reference for the construction of microbial cell factories. We also highlight the current state and problems encountered in the fermentative production of vitamins.

## Introduction

Vitamins are essential for proper growth and health of animals, that cannot produce vitamins by themselves or that synthesize insufficient amount to cover all their needs (Capone and Sentongo, 2019; Suter, 2020). The methods of producing vitamins are based either on chemical synthesis or fermentative production (Yuan et al., 2020).

There are at least 30 kinds of different compounds considered “vitamins,” more than 20 vitamins of which are known to be necessary for biological health. Vitamins are either water-soluble or fat-soluble. As the name suggests, a water-soluble vitamin dissolves in water easily and insoluble in organic solvents. After absorption, the body stores very little of such proteins, and most are excreted with urine (Berdanier and Adkins, 2019). Fat-soluble vitamins are dissolved in fats but not in water, and which are stored in the liver or fatty tissues for future use. While vitamins are essential nutrients for all living things, many plants and microorganisms can synthesize them naturally by themselves. By contrast, humans and other animals need to acquire sufficient vitamins with their diet or through supplements to maintain optimal health (Blake and Konings, 2019).

Traditionally, vitamin production strains have been improved through mutagenesis and metabolic engineering, which can be conducted either through chemical or biological means (Vandamme and Revuelta, 2016b). The main chemical strategies include chemical mutagenesis, application of N^+^ ion beam, ultraviolet radiation or laser mutagenesis. The biological methods mainly include the construction and mutagenesis of the starting strain, genetic modification, synthetic biotechnology, optimization of media and culture conditions, construction of biofilm reactors, etc. (Nie et al., 2013; Song et al., 2014). A series of biotechnological methods are used to transform the metabolic network of cells to construct a programmable “chassis” and “programmable” whole, which can be used to develop an effective assembly strategy, test the adaptability of external components and modules after loading, forming a fine-tuned and customized biological application system. To drive the iterative evolution of other industrial strains, and effectively promote the transformation and renewal of high vitamin producing strains. Chemical methods are usually expensive, environment-unfriendly, waste-prone, and the costly waste disposal. However, the microbial fermentation method has attracted much attention due to low cost, low energy consumption and easy waste recycling. At present, the fermentation method has been recognized by researchers, and it is more environment-friendly and safe than chemical methods. As the fermentation technology matures, this approach is increasingly being used in industry to increase the production of different vitamins. For example, fermentation processes for the production of vitamin B_2_ (VB_2_), vitamin B_12_ (VB_12_), vitamin C, and vitamin K2 have all been industrialized successfully.

Acevedo-Rocha et al. (2019) reviewed the fermentation of B vitamins from the aspect of sustainability. In this review, we mainly discuss vitamins that can be produced by green fermentation processes. It covers water-soluble vitamins, including vitamin C and vitamin B complex (thiamine, riboflavin, niacin, pantothenic acid, pyridoxine, biotin, folate, and cobalamin) as well as the fat-soluble vitamin E and vitamin K. Here, we discussed the producing microorganisms, advanced biological methods and metabolic bottlenecks of different vitamins.

## Water-Soluble Vitamins

### B Vitamins

The global demand for B vitamins is growing due to wide applications in food, pharmaceuticals, feed, and other fields. Although most vitamins are manufactured by chemical synthesis, successful industrial bioprocesses have been established for the production of VB_2_ and VB_12_. The underlying extraordinary achievement in metabolic engineering is discussed in this article.

#### Vitamin B_1_

Vitamin B_1_, which is also known as thiamine, was the first B vitamin to be identified. Thiamine pyrophosphate (TPP), the active form of thiamine, can inhibit the activity of cholinesterase, reduce skin inflammation, prevent seborrheic dermatitis, or eczema, and improve skin health. Thiamine biosynthesis results from the coupling of the pyrimidine and the thiazole moieties to form thiamine phosphate (Dorrestein et al., 2004; Jurgenson et al., 2009; Cea et al., 2020). *Escherichia coli, Salmonella typhimurium*, and *Bacillus subtilis* are the most thoroughly studied thiamine production organisms (Begley et al., 1999).

**FIGURE 1:**
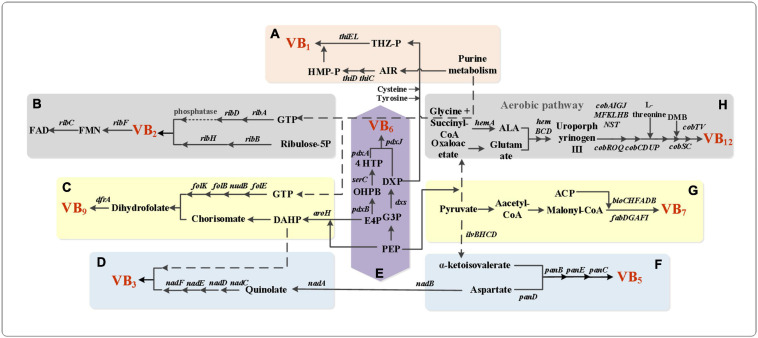
Metabolic network pathway of B vitamins. **(A)** Biosynthesis pathway of thiamine in *E. coli*. ThiC/ThiD, phosphomethylpyrimidine synthase; ThiE, thiamine-phosphate pyrophosphorylase; ThiL, thiamine-monophosphate kinase. **(B)** Biosynthesis pathway of riboflavin in *B. subtilis*. RibA, GTP cyclohydrolase II; RibB, 3,4-dihydroxy 2-butanone 4-phosphate synthase; RibD, diaminohydroxyphosphoribosylaminopyrimidine deaminase; RibH, 6,7-dimethyl-8-ribityllumazine synthase. RibF, FMN adenylyltransferase; RibC, riboflavin synthase. **(C)** Vitamin B_9_ biosynthesis pathway in *B. subtilis*. AroH, chorismate mutase; FolE, GTP cyclohydrolase IA; NudB, dihydroneopterin triphosphate diphosphatase; FolB, 7,8-dihydroneopterin aldolase/epimerase/oxygenase; FolK, 2-amino-4-hydroxy-6-hydroxymethyldihydropteridine diphosphokinase; DfrB, dihydrofolate reductase. **(D)** Vitamin B_3_ biosynthesis pathway in *E. coli*. NadB, L-aspartate oxidase; NadA, quinolinate synthase; NadC, nicotinate-nucleotide pyrophosphorylase; NadD, nicotinate-nucleotide adenylyltransferase; NadE/NadF, NAD + synthase. **(E)** *De novo* biosynthesis pathway of vitamin B_6_. PdxB, erythronate-4-phosphate dehydrogenase; SerC, phosphoserine aminotransferase; PdxA, 4-hydroxythreonine-4-phosphate dehydrogenase; PdxJ, pyridoxine 5-phosphate synthase; Dxs, 1-deoxy-D-xylulose-5-phosphate synthase. **(F)** Pathway for *de novo* synthesis of vitamin B_5_. *ilvBHCD*, increased the transcription levels of the *ilv* genes; *panDBEC*, pantothenate biosynthetic genes. **(G)** Biosynthesis pathway of Vitamin B_7_ in *E. coli*. BioC, malonyl-CoA *O*-methyltransferase; BioH, pimeloyl-[acyl-carrier protein] methyl ester esterase; BioF, 8-amino-7-oxononanoate synthase; BioA, 8-amino-7-oxononanoate aminotransferase; BioD, dethiobiotin synthetase; BioB, biotin synthase; FabD, *S*-malonyltransferase; FabG, 3-oxoacyl-(acyl-carrier protein) reductase; FabA, 3-hydroxyacyl-(acyl-carrier protein) dehydratase; FabF, 3-oxoacyl-(acyl-carrier-protein) synthase II; FabI, enoyl-(acyl-carrier protein) reductase I. **(H)** The aerobic pathway in the synthesis pathway of cobalamin. HemA, glutamyl-tRNA reductase; ALA, δ-aminolevulinate; HemB, porphobilinogen synthase; HemC, hydroxymethylbilane synthase; HemD, uroporphyrinogen-III synthase; CobA, uroporphyrin-III C-methyltransferase; CobI, precorrin-2 C(20)-methyltransferase; CobG, precorrin-3B synthase; CobJ, precorrin-3B C17-methyltransferase; CobF, precorrin-6A synthase; CobK, precorrin-6A/cobalt-precorrin-6A reductase; CobL, precorrin-6B methyltransferase; CobH, precorrin-8X/cobalt-precorrin-8 methylmutase; CobB, cobyrinic acid a,c-diamide synthase; cobNST, hydrogenobyrinic-acid-a,c-diamide:cobalt cobalt-ligase; CobR, cob(II)yrinic acid a,c-diamide reductase; CobO/CobP, corrinoid adenosyltransferase; CobQ, adenosylcobyric acid synthase; CobS/CobV, adenosylcobinamide-GDP ribazoletransferase; CobC, cobalamin biosynthesis protein; CobD, adenosylcobinamide-phosphate synthase; CobU/CobT, nicotinate-nucleotide–dimethylbenzimidazole phosphoribosyltransferase.

In chassis cell *S. typhimurium*, the thiamine pyrimidine moiety can be produced through *de novo* purine biosynthesis or independently of the *purF* gene through the alternative pyrimidine biosynthesis (APB) pathway (Downs and Roth, 1991; Downs, 1992). According to the phenotypic characteristics of the *abpA* mutant, follow-up studies concluded that the functional APB pathway is essential for thiamine synthesis when *S. typhimurium* grows in the presence of exogenous purines (Downs and Petersen, 1994). Research has shown that overexpression of *thiA*, *nmtA*, and *thiP* in *Aspergillus oryzae* can increase the vitamin B_1_ yield fourfold compared to the wild-type (Tokui et al., 2011). Based on the riboswitch mechanism, mutations in the genes of thiamine pyrophosphate kinase activity (*thiN*) and thiamine-related transport proteins (YkoD and YuaJ) were introduced in *B. subtilis* TH95. It was recently reported that thiamine biosynthesis is strictly regulated by TPP riboswitches in bacteria/eukaryotes and transcriptional repressors in archaea (Hwang et al., 2017). *E. coli* has emerged as the preferred cell factory for TPP production after a riboswitch-based biosensors enabled the discovery of thiamine transporters, combined with overexpression of the native *thiFSGHCE* and *thiD* genes, which are closely related to Fe-S metabolism (Figure 1A and Table 1; Cardinale et al., 2017).

**TABLE 1:** Water-soluble vitamins produced by biotechnological methods.

Vitamins	Strains	Biotechnological method	Medium and precursor	Yield	References
Vitamin B_1_	*B. subtilis* TH95	Mutation of gene encoding thiamine pyrophosphate kinase activity (*thiN*) and thiamine-related transport protein (*ykoD* and *yuaJ*).	MM	1.27 mg/L	Schyns et al., 2005
	*E. coli*	TPP biosensor (plasmid pTPP_Bios); Overexpression of native *thiFSGH*; *thiC*; *thiE*; and *thiD*; Genetic-metabolic coupling.	MM	0.80 mg/L	Cardinale et al., 2017
	*A. oryzae*	Overexpression of *thiP*, *thiA*, and *nmtA*.	CD-Dex medium (5% dextrin)	4-fold > WT	Tokui et al., 2011
Vitamin B_2_	*B. subtilis*	Decrease the activity of flavinase RibCF activity; Overexpression of riboflavin biosynthetic genes; improved the *de novo* purine synthesis and pentose supply.	MM	>26 g/L	Schwechheimer et al., 2016
	*A. gossypii*	Introduced the *icl* gene; Overexpression of *gly1*, *prs2,4*, and *prs3* genes; Knocked out *vma4*, *shm2*, and *bas1* genes.	YPD; Plant oil	>20 g/L	Abbas and Sibirny, 2011
	*Candida famata*	Conventional mutagenesis by overexpression of *sef1* and *imh3*.	YPD; Fluorophenilalanine	1026 ± 50 mg/L	Dmytruk et al., 2011
Vitamin B_3_	*Yeast*	Knock out NR importer Nrt1in the NR-non-salvaging genotype *nrkl*, *urhl*, *pmpl* (strain PAB038).	2x YPD; Nicotinic acid	8 mg/L	Belenky et al., 2011
	*E. coli*	Expressing *R. hodochrous* nitrile hydratase.	LB medium; 2YT medium	508 g/L	Wang et al., 2017
Vitamin B_5_	*C. glutamicum*	Deletion of *ilvA* gene and overexpression of *ilvBNCD* and *panBC* genes	MM	1000 mg/L	Leonardi and Jackowski, 2007
	*B. subtilis*	Overexpression of *ilvBHCD* and *panBCDE*; Overexpression of SerA and GlyA of the enzymes of the glycine cleavage cycle.	MM	82–86 g/L	Hohmann et al., 2016
Vitamin B_6_	*E. coli*	Overexpression of native Epd, PdxJ, and Dxs enzymes	MM	78 mg/L	Hoshino et al., 2004
	*S. meliloti* IFO14782	Overexpression of *E. coli* Epd and native PdxJ enzyme.	MM	1.30 g/L	Hoshino et al., 2007
	*B. subtilis*	Overexpression of *E. coli* PdxA and *S. meliloti* PdxJ enzymes.	MM	65 mg/L	Commichau et al., 2015
Vitamin B_7_	*Agrobacterium*/ *Rhizobium* HK4	Overexpression of a strong biotin operon from *E. coli*; Use of a powerful artificial tac promoter and introduct of a modified RBS in front of BioB.	MM; Betaine; Diaminononanoic acid	110 mg/L	Streit and Entcheva, 2003
	*E. coli*	Overexpression of native biotin operon from a high-copy number plasmid	MM; H-medium	11 mg/L	Ifuku et al., 1995
	*B. subtilis*	Overexpression of native biotin operon and selection on *S*-2-aminoethyl-L-cysteine.	MM	21 mg/L	Van Arsdell et al., 2005
Vitamin B_9_	*A. gossypii* (ATCC 10895)	Overexpression of *FOL* genes and deletion of *AgMEY7*; Deletion of *AgADE12* and *AgRIB1* at the same time.	MA2 rich medium	7 mg/L	Serrano-Amatriain et al., 2016
Vitamin B_12_	*S. meliloti* (MC5-2)	High throughput screening of mutants using riboswitch ARTP-irradiation was used to induce random mutations; Deletion of *cobI*; Overexpression of *hemE*.	MM; Cobalt chloride; DMBI	156 ± 4.20 mg/L	Cai et al., 2018
	*P. denitrificans*	Random mutagenesis and genetic engineering; Overexpression of *cobF-cobM* gene cluster and *cogA* and *cobE* genes; Optimize the best PH range; Optimize promoters.	Betaine; Beet molasses; Choline chloride	214.30 mg/L	Li et al., 2008
	*E. coli*	Heterologously expressed the *hemO*, *hemB*, *hemC*, and *hemD* genes etc.; Optimizing of fermentation conditions.	CM medium	0.67 mg/L	Fang et al., 2018
	*Propionibacterium shermanii*	Overexpression of biosynthetic genes.	MM; DMBI	206 mg/L	Sych et al., 2016
Vitamin C	*S. cerevisiae and Zygosaccharomyces bailii*	Overexpressing the endogenous D-arabinono-1,4-lactone oxidase and L-galactose dehydrogenase (overexpression of *lgdh* and *alo1*).	MM	100 mg/L	Sauer et al., 2004
	*K. vulgare* DSM 4025	Oxidation and lactonization.	L/D-sorbose; Glycerol; Baker’s yeast	1.37 g/L	Sugisawa et al., 2005
	*X. campestris* 2286	Lactonation under oxidative stress; Direct synthesis of glucose (carbohydrate source) induced by free radicals (HOCL treatment).	MM; K_2_HPO_4_; Urea	20.40 g/L	Rao and Sureshkumar, 2000
	*G. oxydans* and *K. vulgare* and *B. endophyticus*	Cell–cell interaction; One step 2-KGA fermentation.	D-sorbitol	73.70 g/L (2-KGA)	Ma et al., 2019

*MM, minimal media. CM, complete medium. YPD, yeast extract peptone dextrose. LB medium, Luria-Bertani medium. 2YT medium, Yeast extract and tryptone. CM medium, Cramer-Myers medium. DMBI, 5,6-dimethylbenzimidazole. YD, yeast extract and glucose. MYGP, malt extract, yeast extract, peptone, agar and dextrose.*

**FIGURE 2:**
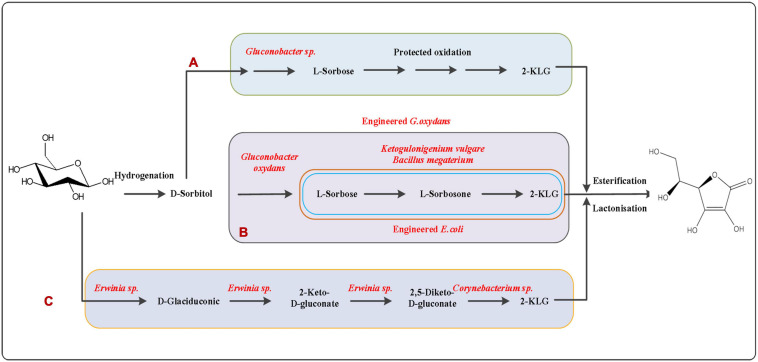
L-ascorbic acid production pathway. **(A)** Classical Reichstein process: the seven-step Reichstein process involving six chemical transformations and one fermentation by *Gluconobacter oxydans*. **(B)** Two steps fermentation by mixed bacteria: *G. oxydans*, *Bacillus megaterium* and *Ketogulonicigenium vulgare*; **(C)** 2,5-Diketo-D-gluconic acid pathway: mixed or two stage fermentations with *Erwinia* sp. and *Corynebacterium* sp. or other species for the direct synthesis of 2-KLG.

However, *thiC/thiH* in the thiamine biosynthetic pathway is involved in Fe–S metabolism and is inhibited by *S*-adenosylmethionine (SAM) metabolites, and the catalytic activity of ThiC enzyme (Figure 2) is very low (*k*_cat_ = 0.002 s^–1^) which is one of the main metabolic bottlenecks (Palmer and Downs, 2013). In addition, the cost of chemical production of thiamine is very low, and the production of engineered strains needs to be increased to be expected to be industrialized.

#### Vitamin B_2_

Riboflavin is an important precursor of flavin mononucleotide (FMN) and flavin adenine dinucleotide (FAD) (Balasubramaniam et al., 2019; Andreieva et al., 2020). Riboflavin insufficiency manifests as persistent anemia (Shi et al., 2014). The biosynthesis of riboflavin begins with guanosine triphosphate and ribose-5-phosphate, followed by six enzymatic steps (Fischer and Bacher, 2005). Burgess et al. (2004) found that overexpression of the *ribABCGH* genes can increase riboflavin production. Later, it was found that there were both nucleotide substitutions and deletions in the regulatory region of the *rib* operon. By deregulating the *rib* operon and purine pathway of *B. subtilis*, riboflavin production was greatly improved. The specific genetic engineering steps included overexpression of the *ribA* gene and deletion of the *purR* gene, after which maximum output of riboflavin reached more than 826.52 mg/L (Figure 1B; Shi et al., 2014). In *Candida famata* overexpression of *sef1* and *imh3* was combined with classic mutagenesis methods to construct the high riboflavin-producing strain AF-4. As a result, 1026 ± 50 mg/L of riboflavin can be produced during a fed-batch cultivation in a lab-scale fermenter. This research has made a great contribution to industrial production of riboflavin (Dmytruk et al., 2011, 2014). The two most important industrial producers are *Ashbya gossypii* and *B. subtilis*.

In *A. gossypii*, malate synthase in the glyoxylate cycle is essential for riboflavin production. Deletion of the malate synthase gene (*ACR268C*) decreased riboflavin production 10-fold compared to the wild-type strain. Conversely, overexpression of the *ACR286C* gene significantly increased the yield of riboflavin by 70%. These results demonstrated that malate synthase is a new target for improving the production of riboflavin (Sugimoto et al., 2009). Abbas and Sibirny (2011) introduced the *icl* gene, overexpressed the *gly1*, *prs2,4*, and *prs3* genes, as well as knocking out the *vma4*, *shm2*, and *bas1* genes, resulting in riboflavin production of more than 20 g/L.

In *B. subtilis*, Schwechheimer et al. (2016) overexpressed riboflavin biosynthesis genes, decreased the activity of the flavin kinase RibCF, and improved the *de novo* purine synthesis and pentose supply, after which the riboflavin yield reached more than 26 g/L. At present, the bottleneck of riboflavin production is mainly due to the poor genetic stability of the engineered strain, and more by-products produced by fermentation, which restrict the high yield of riboflavin.

#### Vitamin B_3_

Niacin is the precursor in the synthesis of the pyridine coenzymes NAD (nicotinamide adenine dinucleotide) and NADP (nicotinamide adenine dinucleotide phosphate) (Chand and Savitri, 2016; Chauhan and Poddar, 2019; Tannous et al., 2020). It is found at relatively high concentrations in internal organs of animal, muscle tissues, and fruits. Currently, niacin is mainly used as a feed additive to increase the utilization of feed protein, or as a pharmaceutical intermediate in the synthesis of various drugs. So far, there is no systematic description of a commercial fermentation process of nicotinic acid (NA) or nicotinamide (NAM). Industrial production methods are mainly ammonia oxidation and electrolytic oxidation, but the former has high production costs and needs to be above 300°C during the reaction, and the latter has low costs of production, however, the efficiency of electrolysis is not high, which limits the industrial production of niacin (Chand and Savitri, 2016).

Recent reports describe the use of recombinant *E. coli* expressing *Rhodococcus rhodochrous* nitrile hydratase for vitamin B_3_ production. At low cell density, nicotinamide was produced in fed-batch mode, and the product concentration reached 390 g/L. After high-density culture in 5 L bioreactor, the concentration of nicotinamide reached 508 g/L in 60 min (Figure 1D; Wang et al., 2017). Belenky et al. (2011) showed that the disruption of *nrt1* results in increased export of nicotinamide riboside (NR). Moreover, disruption of the niacin transporter Tna1 can also increase the output of niacin, revealing that cells regulate the intracellular NAD^+^ metabolic process by balancing the transport of niacin, the precursor of NAD^+^. On the basis of adding 5 mM niacin, yeast cells can produce 8 mg/L nicotinamide mononucleotide (Belenky et al., 2011).

#### Vitamin B_5_

Vitamin B_5_, also known as pantothenic acid, is composed of pantoic acid and β-alanine (β-Ala), which is a precursor of coenzyme A (Leonardi and Jackowski, 2007). It plays an important role in maintaining the health of skin and blood. Its general function is to participate in the production of energy in the body, but it can also control the fat metabolism, and is also an essential nutrient for the brain and nerves. There are chemical and microbial synthesis methods for the synthesis of pantothenic acid, whereby microbial methods can be used to directly synthesize optically pure D-pantothenic acid.

Sahm and Eggeling (1999) adopted a series of methods to increase the production of pantothenic acid, including the deletion of the *ilvA* gene and the overexpression of the *ilvBNCD* and *panBC* genes. The pantothenic acid production of the best strain reached 1000 mg/L (Figure 1F; Sahm and Eggeling, 1999). Huser et al. (2005) also used *Corynebacterium glutamicum* to produce pantothenic acid. They deleted the *ilvA* gene, inhibited the expression of the *ilvE* gene and overexpressed the *ilvBNCD* gene. The final titer of pantothenate reached 1.75 g/L (Huser et al., 2005). Studies have shown that the specific activity of pantothenic acid synthase PanC of *C. glutamicum* is 205.10 U/mg. Adding substrates (D-pantothenic acid and β-Ala) to *E. coli* containing the enzyme can be produced 97.10 U/mg within 32 h, the conversion rate of pantothenic acid was 99.10%. However, the reported work had production defects, which required the addition of exogenous substrate pantothenic acid, and the high market price of pantothenic acid seriously restricted the industrialization of this method. Another chassis organism that is commonly used to produce pantothenic acid is *B. subtilis*. Hohmann et al. (2016) clarified the highest production of pantothenic acid by overexpressing *ilvBHCD*, *panBCDE*, *serA*, and *glyA*, as well as the enzymes of the glycine cleavage cycle the purpose is to increase the number of precursors for pantothenic acid synthesis (Figure 1F). The maximal output of the best strain reached 82–86 g/L during a 48 h fed-batch fermentation, opening up a new chapter of vitamin production in the biological world (Hohmann et al., 2016).

#### Vitamin B_6_

There are six forms of vitamin B_6_, including pyridoxine (PN), pyridoxal (PL), and pyridoxamine (PM), as well as their respective phosphate derivatives. It is a water-soluble vitamin, which exists in the form of phosphate in the body. The most versatile form of vitamin B_6_ is pyridoxal 5′-phosphate (PLP), which is a cofactor of many proteins and enzymes in all organisms. As the most widely available commercial form, PN hydrochloride is extensively used in the pharmaceutical and food industries (Eliot and Kirsch, 2004).

Two *de novo* synthesis routes have been reported the 1-deoxyxylulose 5-phosphate (DXP)-dependent pathway and the DXP-independent pathway (Tanaka et al., 2005). From large-scale screening studies of different strains, found that the Gram-negative bacterium *Sinorhizobium meliloti* is the best producer of vitamin B_6_, reaching a titer of 103 mg/L of B_6_ isoforms within 168 h. Vitamin B_6_ production was further increased to 1.30 g/L by expressing the *E. coli epd* gene and the native *dxs* gene in this *S. meliloti s*train (Figure 1E; Hoshino et al., 2007). *E. coli* and *B. subtilis* were also engineered to produce vitamin B_6_. The vitamin B_6_ production was enhanced to 78 mg/L within 31 h in *E. coli*, and *B. subtilis* produced 65 mg/L of PN when supplied with the precursor 4-hydroxy-L-threonine (4HT) (Hoshino et al., 2004). At present, the industry mainly adopts the oxazole method to produce vitamin B_6_, and the current research also focuses on the improvement of the oxazole method synthesis process. In the process of biosynthesis, the PdxJ enzyme activity is very low (*k*_cat_ = 0.07 s^–1^), and the reaction step catalyzed by this enzyme is the rate-limiting step in the VB_6_ biosynthetic pathway. The intermediate metabolite 4-phosphate hydroxy-threonine (4HTP) is cytotoxic and is also the main bottleneck of biosynthesis. Therefore, the fermentative production of vitamin B_6_ requires more effort to meet the commercial demand.

#### Vitamin B_7_

Biotin is indispensable for the normal metabolism of fats and proteins (Lin and Cronan, 2011; Selvam et al., 2019). It is a nutrient necessary for human growth, development and normal function. Biotin combines with enzymes to participate in the process of carbon dioxide fixation and carboxylation in the body. The current large-scale production of D-biotin is mainly based on the Sternbach synthetic route, and the current industrial production method were improved on this basis. Unless biosynthetic methods can obtain high output at low cost, it is difficult to shake the position of chemical synthesis technology in industrial production. Nevertheless, it was recently reported that some microorganisms can overproduce biotin, which has been elaborated in *C. glutamicum, Mesorhizobium loti*, and *S. meliloti*.

In *Agrobacterium* and *Rhizobium* HK40, overexpression of the biotin operon from *E. coli* driven by the powerful *tac* promoter and introducing a modified RBS in front of *bioB* resulted in a biotin yield of 110 mg/L (Figure 1G; Streit and Entcheva, 2003). If the native biotin operon is overexpressed in *B. subtilis*, most enzymes will be strongly inhibited by the by-product of SAM. However, the high demand for SAM by biotin synthase and 7,8-diaminononanoate synthase is still a bottleneck that must be addressed in future research. If lysine is supplied to *B. subtilis*, BioK will use lysine as the amino donor of the biotin precursor to promote the production of biotin precursor (dephosphorization biotin), and the fermentation process used carbon-limited fed-batch growth conditions with computer control of dissolved oxygen concentrations, but the maximal titer can only reach 21 mg/L biotin. Therefore, improving the catalytic mechanism of biotin synthase is also a challenge for future research (Van Arsdell et al., 2005; Lin and Cronan, 2011).

#### Vitamin B_9_

Naturally occurring folic acid is mostly found in the form of polyglutamic acid, and the biologically active form of folic acid is tetrahydrofolate (Myszczyszyn et al., 2019). Deficiency can lead to reduced hemoglobin content in red blood cells, impaired cell maturation and megaloblastic anemia (Lucock, 2000). *B. subtilis* or *A. gossypii* were successfully engineered to produce folic acid.

Jagerstad and Jastrebova (2013) achieved a 5-methyltetrahydrofolate (THF) titer of 0.95 mg/L by increasing the supply of precursor substances and blocking the catabolic pathway of THF in *B. subtilis*. With the continuous research progress, *A. gossypii* has attracted increasing interest as the chassis strain for folic acid production. *A. gossypii* can synthesize 0.04 mg/L of folic acid naturally, which can reach 6.59 mg/L after metabolic engineering treatment. This is also the highest production value reported to date (Figure 1C; Serrano-Amatriain et al., 2016). Since the commercial chemical synthesis of folic acid is cheap, unless the environmentally unfriendly part of the chemical synthesis process is restricted, there is still a long way to go for the fermentation of this product.

#### Vitamin B_12_

Cobalamin is the only vitamin containing metal elements. Cobalamin is the general term for a class of corrin compounds containing cobalt (Osman et al., 2021). It is the largest and most complex vitamin molecule discovered so far. Vitamin B_12_ deficiency leads to increased formation of ring sideroblasts in pre-myelodysplastic syndromes (Kitago et al., 2020).

Vitamin B_12_ is synthesized by microorganisms through *de novo* synthesis or salvage synthesis in nature, but higher-animals and plants cannot produce it (Figure 1H; Fang et al., 2017). Although in the 19th century, researchers have completed the full chemical synthesis of vitamin B_12_, the chemical synthesis method is too complicated and expensive, so the world’s major suppliers rely on microbial fermentation to produce vitamins. *Pseudomonas denitrificans* and *Propionibacterium freudenreichii* being widely used in industrial fermentation to produce vitamin B_12_. In order to improve the productivity of vitamin B_12_, researchers have adopted a random mutagenesis method to construct a vitamin B_12_ overproducing strains by ultraviolet rays, nitrosoguanidine (NTG), nitrosomethylurethane and ethyleneimine (Blanche et al., 1992, 1995a,b). *Propionibacterium shermanii* was reported to produce vitamin B_12_ with a maximum titer of 200 mg/L (Sych et al., 2016). However, the aerobic *P. denitrificans* remains the most used industrial host, and the effect is obvious. Moreover, *P. denitrificans* has stronger production capacity than the anaerobic strain that produces vitamin B_12_ and is widely used in industrial production. Xia et al. (2015) increased the production of vitamin B_12_ to 198 ± 4.60 mg/L by optimizing the fermentation medium using response surface method. Our research group used *E. coli* strain MG1655 (DE3) as the starting strain to achieve *de novo* synthesis of vitamin B_12_ (Fang et al., 2018). Cai et al. (2018) later used riboswitch elements in *S. meliloti* for the first time, and successfully developed a flow cytometry high-throughput screening system for high-yield VB_12_ strains. The vitamin B_12_ titer of the best strain, *S. meliloti* MC5–2, reached 156 ± 4.20 mg/L, but the yield was still relatively low. At the same time, they also emphasized that the titer of vitamin B_12_ is greatly dependent on the medium composition (Cai et al., 2018).

### Vitamin C

Vitamin C, also known as L-ascorbic acid (LAA), is an important cofactor for multiple enzyme reaction in the body (Paciolla et al., 2019; Kawahori et al., 2020). It can act as an antioxidant to scavenge free radicals and reduce oxidative stress, so a rapidly expanding market is the application of LAA as an additive to cosmetic products (Timoshnikov et al., 2020). Vitamin C deficiency can result in scurvy. Recently, researchers used biochemical methods combined with DNA recombination technology to produce vitamin C.

At present, L-AA is commercially manufactured via the classic seven-step Reichstein process using D-glucose as the initial substrate. The process involves six chemical steps and one fermentation steps for the oxidation of D-sorbitol to 2-keto-L-gulonic acid (2-KGA) by *Gluconobacter oxydans* and *Bacillus megaterium* (Figure 2A). Sugisawa et al. (2005) reported for the first time that *Ketogulonigenium vulgare* DSM 4025 can produce 1.37 g/L of L-AA under static culture conditions. Kim et al. (1996, 1998) reported that the respective enzymes from *Candida albicans* and *S. cerevisiae* convert not only D-arabinose to D-arabinono-1,4-lactone but also L-galactose to L-galactono-1,4-lactone *in vitro*. Experiments have shown that budding yeast cells overexpressing the endogenous D-arabinono-1,4-lactone oxidase and L-galactose dehydroge-nase can produce about 100 mg/L of L-ascorbic acid (Sauer et al., 2004). A microbiological consortium composed of *G. oxydans*, *K. vulgare*, and *B. endophyticus* was constructed to produce 2-KGA, and a final yield of 73.70 g/L was obtained within 30 h (Figure 2B; Ma et al., 2019). This result holds promise for the construction of a microbial cell factory for the production of vitamin C. However, it has been reported that mixed-bacteria fermentation can be unstable due to competition between the individual strains for nutrients and other factors. Therefore, mixed-bacteria fermentation technology has poor stability and low efficiency, which also hinders the pace of industrial production of vitamin C. Nevertheless, fermentation is expected to become the mainstream way of vitamin C production in the future if stable single strains can be used instead of mixed bacteria fermentation, while also shortening the production cycle.

## Fat-Soluble Vitamins

### Vitamin A

Vitamin A mainly includes β-carotene, α-carotene, and β-cryptoxanthin (Wise et al., 2021). β-carotene, a provitamin A carotenoid, is divided into all-*trans* and *cis* isomers (Yang et al., 2021). All-*trans*-β-carotene is the major isomer found in unprocessed carotene-rich plant foods, followed by its 9- and 13-*cis* isomers. β-carotene is an antioxidant, which not only inhibits singlet oxygen but also inhibits lipid peroxidation, thereby playing an important role in the prevention of disease (Kawata et al., 2018).

**FIGURE 3:**
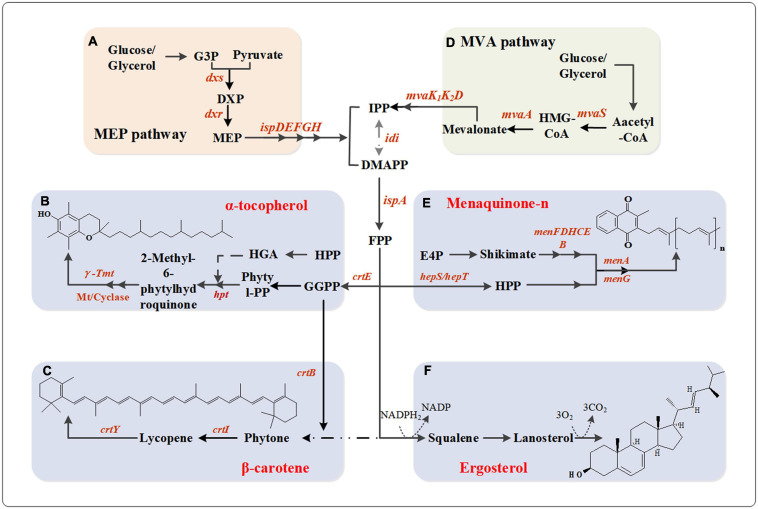
Metabolic network pathway of vitamin A/D/E and vitamin K. **(A)** MEP pathway. Dxs, 1-deoxy-D-xylulose-5-phosphate synthase; Dxr, 1-deoxy-D-xylulose-5-phosphate reductoisomerase; IspD, 2-*C*-methyl-D-erythritol 4-phosphate cytidylyltransferase; IspE, 4-diphosphocytidyl-2-*C*-methyl-D-erythritol kinase; IspF, 2-*C*-methyl-D-erythritol 2,4-cyclodiphosphate synthase; IspG, 1-hydroxy-2-methyl-2-(*E*)-butenyl 4-diphosphate synthase; IspH, 4-hydroxy-3-methylbut-2-enyl diphosphate reductase; **(B)** α-tocopherol biosynthesis pathway. Idi, isopentenyl diphosphate isomerase; IspA, geranyltranstransferase; CrtE, GGPP synthase; hpt, hypoxanthine phosphoribosyltransferase; γ-Tmt, γ-tocopherol methyl-transferase; Mt, methyl-transferase. **(C)** β-carotene biosynthesis pathway. CrtB, phytoene synthase; CrtI, phytoene desaturase; CrtY, lycopene cyclase. **(D)** MVA pathway. MvaS, HMG-CoA synthase; MvaA, HMG-CoA reductase; MvaK1, mevalonate kinase; MvaK2, phosphomevalonate kinase; MvaD, diphosphomevalonate decarboxylase; **(E)** Menaquinone-*n* biosynthesis pathway. HepS/HepT, heptaprenyl diphosphate synthase component I/II; MenF, isochorismate synthase; MenD, 2-succinyl-5-enolpyruvyl-6-hydroxy-3-cyclohexene-1-carboxylate synthase; MenH, demethylmenaquinone methyltransferase; MenC, *o*-succinylbenzoate synthase; MenE, *o*-succinylbenzoate-CoA ligase; MenB, 1,4-dihydroxy-2-naphthoyl-CoA synthase; MenA, 1,4-dihydroxy-2-naphthoate heptaprenyltransferase; MenG, demethylmenaquinone methyltransferase. **(F)** Ergosterol biosynthesis pathway.

Carotene is mainly produced by fungi, some bacteria, and algae. For example, Yoon et al. increased the supply of IPP (isopentenyl diphosphate) and DMAPP (dimethylallyl diphosphate) through the introduction of foreign MVA (mevalonate) pathway (Figure 3D), thereby enhancing the production of carotenoids. The final engineered *E. coli* with a whole MVA pathway and β-carotene synthesis gene can produce β-carotene of 465 mg/L (Figure 3C; Yoon et al., 2009). Adenosine-triphosphate (ATP) and nicotinamide adenine dinucleotide phospha (NADPH) are two important cofactors in β-carotene biosynthesis pathway. Zhao et al. (2013) used *E. coli* as host cells, constructed and optimized a central metabolic module to increase the supply of ATP and NADPH in β- carotene synthesis pathway, thereby improving the yield of the β-carotene. Finally, the best strain CAR005 increased the β-carotene production to 2.1 g/L with a yield of 60 mg/g DCW in fed-batch fermentation (Zhao et al., 2013). Larroude et al. (2018) overexpressed heterologous carotene synthase (Crt) in *Yarrowia lipolytica* to make it produce high β-carotene. The fermentation yield of the engineered strain obtained by screening the best promoter was 1.5 g/L. By optimizing the fermentation conditions and using fed-batch fermentation, the yield of β-carotene was further increased production titer of 6.5 g/L and 90 mg/g DCW (Larroude et al., 2018). However, the insufficient number of precursors seriously hindered the industrialization process of β-carotene in the process of β-carotene synthesis in the future.

### Vitamin D

Vitamin D refers to a group of fat-soluble secosteroids responsible for increasing intestinal absorption of magnesium, calcium, and phosphate, and many other biological effects. The most important compounds are vitamin D_2_ (ergocalciferol) and vitamin D_3_ (cholecalciferol) in vitamin D. Vitamin D can increase intestinal absorption of calcium, magnesium, and phosphate, and can prevent many diseases (Yuan et al., 2020).

It is well known that the precursor of vitamin D_2_ is ergosterol (Papoutsis et al., 2020). Vitamin D_2_ is widely used in medical, food and other industries. The current commercial production of ergosterol is mainly produced by yeast fermentation. Tan et al. improved the production of ergosterol by optimizing the fermentation medium and screening high ergosterol producing strains (Figure 3F). The results show that dissolved oxygen (DO) can be used as the effective control parameter for yeast fed-batch fermentation. The total yield of ergosterol can be increased to 1.16 g/L when DO was controlled at 12% (±1%) and pulse fed-batch was used (Tan et al., 2003).

Vitamin D_3_ cannot play a direct role in human and animals, but it can produce the physiologically active form 25-hydroxyvitamin D_3_ (25-OH-VD_3_) through the metabolism in liver. At present, the production process of 25-OH-VD_3_ mainly includes chemical synthesis and light irradiation. The chemical reaction steps are cumbersome, some of them need halogen reagents, and the racemates are generated during the reaction, which makes the separation difficult. Therefore, more and more researchers pay attention to the fermentation of 25-OH-VD_3_ by microorganisms. The strains used in microbial biosynthesis mainly include *Rhodococcus*, *Streptomyces*, *Pseudonocardia* sp., and *Mycobacterium*. Vitamin D_3_ hydroxylase (Vdh) is a kind of cytochrome P450 monooxygenase, which can catalyze the two-step hydroxylation of vitamin D_3_ (VD_3_) to produce 25-OH-VD_3_ and 1α,25-dihydroxyvitamin D_3_. Yasutake et al. (2013) used nisin, a natural bioactive antimicrobial peptide, to treat *Rhodococcus* cells containing hydroxylase, and they found that 573 mg/L 25-OH-VD_3_ can be synthesized. Although the current industrial production of vitamin D_3_ is mainly dominated by chemical synthesis, microbial synthesis methods are more sustainable and do not produce impurities during the biosynthesis process, thus it will be taken priority in the future industrial production.

### Vitamin E

Vitamin E is a group of lipid-soluble antioxidants, including tocopherols and tocotrienols (Muñoz and Munné-Bosch, 2019; Zeng Z. et al., 2020). These compounds are composed of an oxygen-containing double ring system with a hydrophobic prenyl side chain (Blake and Konings, 2019). Lack of vitamin E affects the function of T and B immune cells (Moriguchi and Muraga, 2000). Additionally, patients with severe impairment due to Alzheimer’s disease improved significantly after receiving α-tocopherol (Sano et al., 1997). Considering various physiological effects of tocopherols, they are widely used in the manufacture of human dietary supplements, food preservatives and cosmetics. There are four different tocopherol compounds, named α, β, γ, and δ tocopherol. Among the four forms of vitamin E, α-tocopherol is the most biologically active (Kaiser et al., 1990). In nature, α-tocopherol is produced by photosynthetic organisms, e.g., eukaryotic algae and green plants, some prokaryotic cyanobacteria, such as *Synechocystis*, which can accumulate vitamin E in large amounts (Figure 3B; Taketomi et al., 1983).

Recently, *Euglena gracilis* was found to be suitable for the production of high-value products, such as amino acids and ascorbic acid (Schwarzhans et al., 2015). *E. gracilis* is the most promising host for the commercial production of α-tocopherol, with a high growth rate and α-tocopherol content, which accounts for more than 97% of the total tocopherol accumulated by *E. gracilis*. Tani and Tsumura added precursors such as homogentisate and L-tyrosine to *E. gracilis* growth medium, which increased the accumulation of α- tocopherol to 143.60 mg/L corresponding to 5.1 mg/g dry cell weight (DCW) (Tani and Tsumura, 1989). Durmaz (2007). explored the effect of nitrogen source and concentration on the accumulation of α-tocopherol in *Nannochloropsis oculata.* When sodium nitrate and ammonium chloride were used as inorganic nitrogen source, the highest content of α-tocopherol reached 2.32 ± 0.04 mg/g dry weight (DW) (Table 2). The research showed that higher concentrations of nitrogen in the form of NO_3_^+^ and NH_4_^+^ can promote production the of α-tocopherol (Durmaz, 2007).

**TABLE 2:** Fat-soluble vitamins produced by biotechnological methods.

Strain	Biotechnological method	Main culture substances	Yield	References
**Vitamin A**				
*E. coli*	Glycerol as the carbon source and harboring the whole MVA pathway.	2YT medium; Glycerol	465 mg/L	Yoon et al., 2009
*E. coli*	Overexpression of *crt* genes, *dxs*, *idi*, *sucAB*, *sdhABCD*, and *talB*.	LB medium	2.1 g/L	Zhao et al., 2013
*Y. lipolytica*	Expressing the heterologous pathway and screen the best combination of promoters for each of the studied genes.	YPD medium; MM medium; YNB medium	6.5 g/L	Larroude et al., 2018
**Vitamin D**				
*S. cerevisiae*	DO was kept at 12% (±1%) and pulse fed-batch was used.	MM medium	1.16 g/L VD_2_	Tan et al., 2003
*R. erythropolis*	Insert the gene-expression cassette encoding *Bacillus megaterium* glucose dehydrogenase-IV into the chromosome of *R. erythropolis*.	MM medium	573 mg/L VD_3_	Yasutake et al., 2013
**Vitamin E**				
*E. gracilis*	Add effective additives (homogentisate and L-tyrosine); Optimize the concentration of ethanol and protein.	KH medium; Homogentisate; L-tyrosine	5.10 mg/L	Tani and Tsumura, 1989
*E. gracilis*	Determination of the amount of α-tocopherol produced under photoautotrophically, heterotrophically or photoheterotrophically.	MM; Methane	8.60 ± 0.22 mg/L	Grimm et al., 2015
*Stichococcus bacillaris*	Ballon bioreactor culture with MeJa as inducer.	Methyl jasmonate (MeJa); Algal culture	0.60 mg/g (DW)	Sivakumar et al., 2014
*Nannochloropsis oculata*	Optimize the carbon source of the medium (NO3^+^-N and NH4^+^-N) and harvest time.	F/2 medium; Ammonium chloride	2.32 ± 0.04 mg/g (DW)	Durmaz, 2007
*S. cerevisiae*	Gene cloning from various photosynthetic organisms; Codon optimization and protein truncation.	SD medium	320 mg/L	Shen et al., 2020
**Vitamin K (MK-4/MK-7)**				
*B. subtilis natto*	Optimum media conditions and screening producing strain (Different nutrients of the culture medium will affect the yield of MK-7).	Glycerol	62.32 ± 0.34 mg/L	Berenjian et al., 2011
*B. subtilis natto*	Fermentation using soybean extract and screening highest MK7 yielding strain from commercially available natto.	Soy granules; Amylase	67.01 ± 0.18 mg/kg	Mahanama et al., 2011
*B. subtilis*	Deletion of *PAS-A, kinB, spoIIA, spo0IIE, dhbB, and ptsG;* Overexpresion of *menF*, *menB*, *menE*, *entC*, *ppsA*, *aroK*, *ispA*, *hepS/T*, *kdpG*, *dxr*, *dxs*, *fni*, *menA*.	LB Medium	200 mg/L	Cui et al., 2019
*B. subtilis*	Overexpresion of *BS*20*- qcrA-C and tatAD-CD.*	LB Medium	410 mg/L	Cui et al., 2020

*YNB medium, yeast nitrogen base; DW, Dry weight; SD medium, synthetic dextrose medium; MYP agar, mannitol, egg yolk and polymyxin agar; TBAB, tetrabutylammonium bromide; CDW, cell dry weight.*

To balance cell growth and product synthesis, Shen et al. (2020) recently combined heterologous genes from photosynthetic organisms with the endogenous shikimate and mevalonate pathways (MEP) to construct a strain of *S. cerevisiae* that produces tocotrienols (Figure 3A). By incorporating a newly designed cold-shock-triggered temperature control system, the phased control of cell biomass and tocotrienol accumulation by the engineered strains was successfully realized. The final total tocotrienol titer reached 320 mg/L in a 5 L fermenter, which laid the foundation for the production of natural vitamin E in a fully fermentative process (Figure 3B; Shen et al., 2020).

In general, compared with chemical total synthesis, the method of obtaining vitamin E directly through biotechnology has low yield and high cost, and is not suitable for large-scale production. Although chemical total synthesis is currently the main production method of vitamin E, there are still many problems with this technology, such as complex synthesis routes, high technical barriers, etc. Therefore, the development of safer and more efficient synthesis technology has become the main problem to improve the current situation of vitamin E.

### Vitamin K

Vitamin K is a fat-soluble vitamin, which also called blood coagulation vitamin in virtue of the function of promoting blood coagulation and preventing osteoporosis (Henrik, 1973; Schwalfenberg, 2017; Zhou et al., 2019). There are two naturally occurring types of vitamin K, called vitamin K1 (phylloquinone/phytomenadione) and vitamin K2 (menaquinone, MK) (Holvik et al., 2019). Vitamin K1 is synthesized by plants, while vitamin K2 is synthesized by microorganisms and can be divided into 14 isoforms depending on the number of isoprenoid units connected to the menaquinone ring (Figure 3E; Schwalfenberg, 2017). Among them, menaquinone-7 (MK-7) is the most effective subtype of vitamin K with a very long half-life in circulation. Notably, MK-7 can be synthesized in the *cis*, *trans*, and *cis/trans* forms, but only the all-*trans* form is biologically active (Szterk et al., 2018).

The biosynthesis of MK-7 from the embden-meyerhof-parnas (EMP) pathway, pentose phosphate pathway (PPP), MVA pathway and menadione synthesis (MK) pathway (Figure 3C). A number of microorganisms have been used to produce MK-7, including *B. subtilis, E. coli*, lactic acid bacteria, *Flavobacterium* sp., and *B. amyloliquefaciens* (Sharma et al., 1993; Morishita et al., 1999; Sato et al., 2001; Wu, 2011; Taguchi et al., 2014).

*Bacillus subtilis* isolated from natto (a traditional Japanese food), the strain found in the eponymous fermented Japanese beans, has been certified by the FDA as a food-safe and has a strong ability to produce MK-7. Accordingly, *B. subtilis natto* was used as a parent strain to develop some of the industrial strains currently on the market. In industrial production, *B. subtilis natto* fermentation broth was sprayed and dried, and the dry powder from the fermentation broth was subjected to solvent extraction. The obtained extract was condensed into a paste and then purified by chromatography. Using this process, the final yield of MK-7 can reach 200–300 mg/L in the fermentation cycle of 16–24 h (Chen et al., 2016).

Cui et al. (2019) developed a bifunctional quorum-sensing system in *B. subtilis* 168 to engineer the synthesis modules of MK-7. The resulting strain was capable of producing 360 mg/L MK-7 in shak flasks and 200 mg/L MK-7 in 15-L bioreactor (Cui et al., 2019). Recently, comparative transcriptomics revealed that cell membrane and electron transfer engineering in *B. subtilis* can improve the synthesis of MK-7. The resulting strain reached a product titer of 410 mg/L after 6 days in shake-flask culture, which is the highest value reported to date (Cui et al., 2020). In the current market environment, the production of natural all-trans MK-7 is via liquid fermentation of *B. subtilis natto*, which is safe, natural and controllable, and occupies the mainstream position in the market. Compared with the chemically synthesized of *trans*-MK-7, it has a higher yield and fewer impurities.

## Conclusion

The fermentative production of vitamins using bacteria, yeasts or microalgae has many advantages over traditional chemical synthesis methods. From the aspects of safety, biological activity, absorption rate, etc., vitamins manufactured by biological methods can be more suitable for both internal and external applications (Yuan et al., 2020). Although the fermentation of VB_2_ and VB_12_ has technologically matured and is being applied in industrial production, fermentation methods for the remaining B-group vitamins have yet to be developed or require significant yield improvement.

Vitamin C has a large market, and its production method is mainly based on single-bacteria fermentation, which eliminates the dependency of associated bacteria by replacing accompanying bacteria with associated active agents (Vandamme and Revuelta, 2016a). However, the current market situation indicates that vitamin C production has overcapacity, the downstream processing is complicated, and the market demand is concentrated in the field of medicine and food. For these reasons, the momentum of price increase will remain slow in the future. Vitamin C fermentation technology can explore the mechanism of a variety of accompanying bacteria, establish their anabolism database, or use isotope technology to label and trace the individual metabolites. It is also possible to design heterologous assembly modules for 2-KGA synthesis, and study adaptation mechanisms in microbial chassis cells, so as to achieve higher productivity (Liu et al., 2011).

The current biosynthesis product of vitamin A is mainly focused on β-carotene. The biosynthesis of β-carotene has successfully established a large-scale production process through classical and reasonable microbial metabolic engineering. However, due to the high barriers of intermediate industry and the complex process of synthesis and metabolism, the future research will face more difficult challenges. At present, the industrial production of vitamin D is mainly through the chemical synthesis of active 25-OH-VD_3_ and 1α,25-dihydroxyvitamin D_3_, but the biggest obstacle is the assured quality and security of supply for raw materials, which must be cholesterol with purity greater than 95% (NF grade). Therefore, the key to solving the problem of raw materials is to develop more production bacteria, optimize their metabolic pathways and make them highly productive. With the continuous optimization and technological progress of vitamin E fermentation, the overall cost of the industry has fallen, which promoted the growth of the industry. Unfortunately, although studies have shown that photosynthetic microorganisms have considerable potential for the production of tocopherols, light-driven fermentation is costly, which makes commercialization difficult. However, due to the considerable potential *E. gracilis* and the conditions of the cultivation environment, the construction of a specifically designed photo-bioreactor may be a feasible research direction for the production of tocopherol. Moreover, the controllable temperature-sensitive control system may also be a key control technology for vitamin E production. Among vitamin K producing bacteria, *B. subtilis natto* seems to be the most promising candidate for MK production. Many researchers have optimized the design of fermentation modes, medium components, and culture conditions. They have also applied genetic engineering and other means to increase MK production (Szterk et al., 2018). However, to achieve higher industrial output, the technology needs to be further improved. Some studies have used biofilm reactors, which may become a promising new area for future research.

Recently, our research group used *E. coli* MG1655 (DE3) as chassis strains and achieved the *de novo* synthesis of vitamin B_12_ via metabolic engineering and optimization of fermentation conditions. In addition, we have not only proved that *E. coli* is a microbial biosynthesis platform for the production of vitamin B_12_, also provides an encouraging example of how the dozens of proteins in a complex biosynthetic pathway can be transferred between organisms to promote industrial production (Fang et al., 2018). In addition, our research group is also doing metabolism research on vitamin B_2_, B_6_, B_7_, and vitamin K.

In general, the development of synthetic biotechnology provides new opportunities for the construction of vitamin cell factories. First, high-throughput screening of high-yield strains, the CRISPR/Cas9 genome editing technology, and automatic gene assembly technology provide important technical means for the mining and genetic modification of chassis cells (Chang et al., 2019; Zeng W. et al., 2020; Zhang and Showalter, 2020). Further, the output of vitamin products in different dimensions will be increased by transforming the complex and multi-enzyme pathways required for the production of vitamins, establishing microbial flora with controllable functions and stability, and application of some advanced engineering technology, such as the cold-shock-triggered temperature control system, dynamic control of gene expression systems, different types of biosensors, cell-free systems and computer-aided design, etc. (Koo et al., 2020; Marucci et al., 2020; Sachsenhauser et al., 2020; Shen et al., 2020; Glasscock et al., 2021). Additionally, the modular and orthogonal strategies are increasingly supporting the construction of vitamin cell factories (Liu et al., 2015). The mining and design of biological components, the assembly and integration of elements and modules, and the optimization and adaptation of the fermentation system are also important for efficient production of vitamins (Santos-Merino et al., 2019). However, there will be many challenges in the field of synthetic biotechnology in the future, including the compatibility between flexible biological systems and rigid engineering systems, or the universality of biological system reconstruction. It will be necessary and important to advance the existing technology, combine it with new strategies, and conduct interdisciplinary research to establish novel microbial cell factories for the industrial fermentation of most vitamins. All in all, we firmly believe that the industrialization of the fermentation production of vitamins is expected to become a broader, safer and more sustainable manufacturing with the continuous advancement of synthetic biotechnology and metabolic engineering.

## Author Contributions

YW and LL: manuscript planning, writing, and revision. ZJ and DZ: manuscript revision and writing. All authors contributed to the article and approved the submitted version.

## Conflict of Interest

The authors declare that the research was conducted in the absence of any commercial or financial relationships that could be construed as a potential conflict of interest.
